# Predictors of Young Adult Women’s Psychological Well-Being in Romantic Relationships

**DOI:** 10.3390/bs15010082

**Published:** 2025-01-18

**Authors:** Elif Yöyen, Süreyya Çalık, Tülay Güneri Barış

**Affiliations:** 1 Department of Psychology, Faculty of Humanities and Social Sciences, Sakarya University, Sakarya 54050, Türkiye; 2 Institute of Social Sciences, Department of Clinical Psychology, Marmara University, İstanbul 34722, Türkiye; 3 Department of Health Sciences, Institute of Business Administration, Sakarya University, Sakarya 54050, Türkiye

**Keywords:** romantic relationship, psychological well-being, empathy, sexual quality of life, relationship stability, passionate love, intimacy in romantic relationships

## Abstract

Psychological well-being is the experience of fewer negative emotions, such as feelings of loneliness, depression, and low mood, and more positive emotions, such as taking pride in one’s life, being enthusiastic about one’s life, and having a highly satisfying purpose in life. In other words, it describes an individual who is happy and free from psychopathology. Psychological well-being in romantic relationships is influenced by several factors, such as empathy, relationship stability, and quality of sex life. This study was conducted to examine the relationship between psychological well-being and empathy, quality of sexual life, love, relationship intimacy, and relationship stability among women in romantic relationships. This study included 415 female participants aged 23–45 who had been in a romantic relationship for at least 12 months. Data were collected using the Sociodemographic Data Form, the Psychological Well-Being Scale (PWBS), the Basic Empathy Scale (BES), the Sexual Quality of Life Scale—Women’s Form (SQLS), the Relationship Stability Scale (RSS), the Passionate Love Scale (PLS) and the Romantic Relationship Closeness Scale (RRCS). Independent group *t*-test, one-way analysis of variance (ANOVA), Pearson correlation analysis, and regression analysis were used to analyse the data. The analyses found that the variables that predicted psychological well-being were the relationship satisfaction sub-dimension of the relationship stability scale, quality of sexual life, empathy, and having children. In addition, correlational analysis revealed that empathy, sexual quality of life, intimacy in romantic relationships, relationship satisfaction, and relationship attachment were significantly positively correlated with psychological well-being. In terms of demographic information, it was found that scores for evaluating options in the relationship increased as education level increased; scores for investing in the relationship increased as education level decreased; passionate love was more common among working women; having children increased psychological well-being but decreased intimacy in romantic relationships; and quality of sexual life increased as seriousness in the relationship increased. The results of this study may be useful to psychologists and psychiatrists in their studies of female clients, to sociologists and public health specialists in their studies of women, and to policy makers in determining public health policies.

## 1. Introduction

Romantic relationships are defined as voluntary interactions that are manifested through mutual expressions of love and sexual behaviour (Collins et al., 2009). Romantic relationships, which consist of a combination of passion, commitment, and closeness between a man and a woman (Sternberg, 1986), arise from the fact that people fundamentally need emotionally positive interactions for long-term, caring relationships (Baumeister & Leary, 2007). Romantic relationships are a biological process designed to facilitate bonding between two adult sexual partners who have a safe and nurturing environment for raising a baby (Gibson, 2023). It is a form of close relationship that results from an individual’s need for a relationship in which closeness, empathy, and attachment to another person lead to feelings of love and closeness (İnanç & Yerlikaya, 2012), support, assistance, respect, attention, and sexual needs are met (Furman & Wehner, 1997).

In this form of close relationship, the concept of closeness is defined as the level of commitment and positive emotional, cognitive, and physical closeness that a person experiences with a partner in a mutual relationship (Moss & Schwebel, 1993). It also includes the comfort and tender feelings of the relationship, as well as the warm component of love (Acker & Davis, 1992). Intimacy can be the heart and soul of a relationship (Prager et al., 2013). The closeness gained in romantic relationships has a significant impact on the interpersonal development, personal harmony, and physical health of individuals (Moss & Schwebel, 1993). Close relationships are enriching and can provide emotional support, love, assurance of care, and a deep understanding of other individuals. Intimacy promotes psychological well-being in individuals. The lack of intimacy, on the other hand, has detrimental effects on the health and psychological well-being of individuals (Prager, 1997). Studies have reported that there is a positive correlation between closeness in women’s romantic relationships and their psychological well-being (Campbell et al., 1994; Debrot et al., 2013; Johnson et al., 2012).

The concept of psychological well-being in romantic relationships has been defined in different ways by different authors: psychological well-being is defined as the experience of less negative affect (feelings of loneliness, depression, and low mood) and more positive affect (pride in one’s life, high aspirations, and satisfaction with one’s life purpose) (Proctor, 2024). Maloney put forward the idea of three ways of conceptualising psychological well-being. The first is the negative model. According to this, psychological well-being is manifest in the absence of psychopathology. He called the second option the normal model. Here, normal people without pathology are people who have a satisfying job, friends, self-perception, have an average level of adjustment, and are defined as happy. According to the last option of the definition of psychological well-being, a psychologically healthy person is defined as someone who has more than an average adjustment to life (Akın, 2009). While Roothman defines psychological well-being as the individual’s state of well-being in terms of emotional, physical, cognitive, spiritual, personal, and social processes (Roothman et al., 2003), Ryff stated that psychological well-being consists of six dimensions (self-acceptance, positive relationships with others, autonomy, ability to cope with the environment, meaning in life, and personal development) (Ryff, 1995). All these dimensions have been presented as a model using the theories of scholars such as Allport, Maslow, Rogers, Erikson, Jung, Maslow, Buhler, Johada and Frankl (Ryff, 2014). Self-acceptance in Ryff’s model is a person’s recognition of many aspects of their self and a positive attitude towards the aspects they recognise. It involves feeling positive about one’s life. It is the individual’s acceptance of many aspects of their self and feeling optimistic about their past. Having positive relationships with others includes understanding the nature of the balance of giving and taking in human relationships and having honest, satisfying, mutually trusting relationships, being interested in the satisfaction of others, having empathy, compassion, and closeness. It is defined as a person who has quality relationships with others (Ryff & Keyes, 1995). Autonomy is explained as thinking and acting in certain ways while evaluating oneself according to personal standards rather than social pressures. It is defined as having an internal focus of evaluation that one creates for oneself rather than turning to others for approval (Ryff, 2014). A person with the capacity to manage the environment is defined as someone who can effectively use the opportunities in his/her environment, choose contexts that are suitable for spiritual needs and values, and benefit from them. The capacity to manage the environment is a future-oriented feature of psychological well-being that emphasises active participation in the environment. A person with purpose in life feels that their current and past lives have meaning. This dimension is explained as a person who has a positive attitude to life and lives a meaningful and purposeful life (Ryff & Keyes, 1995). According to Ryff, a person who functions positively towards this purpose has a sense of direction. This sense of direction contributes to the person’s sense that life is meaningful. Personal development is defined as the individual having a sense of continuous development; seeing themselves developing and changing behaviour; being open to new experiences; having a sense of realising their potential; and showing improvement and change in themselves and their behaviour over time (Ryff, 1989).

Another factor affecting psychological well-being in romantic relationships is the concept of empathy. Empathy is an emotional response that stems from another person’s emotional state and is compatible with that state or emotion (Eisenberg & Strayer, 1990). Empathy is a skill defined as the ability to feel or imagine another person’s emotional experience (McDonald & Messinger, 2011). Empathy is a process involving cognitive and emotional dimensions (Wu et al., 2021). The cognitive dimension of empathy allows one to understand or infer the other’s behaviour and mental states in relationships (Smith, 2006). It is a perceptual process that allows a person to put themselves in the other party’s shoes, imagine, think, and understand what they are feeling (Howe, 2012; Smith, 2006; Wu et al., 2021). The emotional dimension of empathy is defined as the process of being emotionally affected by another person’s situation and experiencing similar feelings despite one’s own, knowing that what is being felt belongs to the other party (Ramlakhan, 2017). The emotional dimension of empathy is the process of empathy felt in the body (Howe, 2012).

Psychological well-being in romantic relationships is also influenced by the quality of sexual life. The quality of sexual life includes the absence of organic diseases and disorders that affect reproduction and sexual function; the absence of fears, shame, guilt, and false beliefs that negatively affect interpersonal relationships; and the ability to control and be satisfied with the pleasure derived from sexual intercourse and sexual behaviour (PAHO, 2000). In fact, sexuality is a fundamental force that can affect every area of life (Arrington et al., 2004). Sexuality is an important element of life for the well-being, success, and connection of the individual with others. Examining the studies on the quality of sexual life and psychological well-being, it is reported that psychological well-being is a strong variable in predicting the satisfaction of sexual life, and especially in terms of the predictors of sexual satisfaction, there are some patterns such as satisfaction with emotional closeness during sexual intercourse, cooperation and satisfaction with sexual intercourse (Dundon & Rellini, 2010). Another study emphasised the need to focus on psychological well-being and harmony in sexual intercourse in addition to psychological or drug treatment in the treatment of sexual dissatisfaction (Haavio-Mannila & Kontula, 1997). In a study conducted with physically disabled people, sexuality and psychological well-being were assessed. It was reported that self-esteem, body image, and depression were strong predictors of sexual satisfaction in physically disabled people (Taleporos & McCabe, 2002). Another study investigating the contribution of psychological well-being to sexual satisfaction reported that the environmental dominance dimension in Ryff’s model was the dimension that contributed most to sexual satisfaction (Biss & Horne, 2005).

Another factor influencing psychological well-being in romantic relationships is the concept of relationship stability. Relationship stability refers to the tendency of individuals in romantic relationships to continue their current relationships, taking into account the satisfaction they receive from the relationship, the level of evaluation of the quality of their options, and the investment they make in the relationship (Büyükşahin et al., 2005). Research on relationship stability focuses on the dimensions of relationship satisfaction, investment in the relationship, evaluation of the quality of options, and commitment. These studies have reported that unstable relationships increase couples’ depressive symptoms and lead to low life satisfaction (Brown, 2000; Drigotas et al., 1999; Robertson et al., 2018).

Another factor influencing psychological well-being in romantic relationships is the concept of love. Schopenhauer explained love as a personalised sexual instinct (Schopenhauer, 2009); Freud explained love as the pursuit of an ego ideal (Sternberg, 1986). Hatfield divided love into two types: passionate love and companionate love (Hatfield, 1982). According to Hatfield and Rapson, companionate love is defined as feelings of affection and tenderness towards intimate people in our lives. Passionate love is described as lovesickness or obsessive love (Hatfield & Rapson, 1993). However, Hatfield describe passionate love as a physiological arousal that includes sensitive, sexual feelings and excitement for union with another person. The desire for union consists of cognitive, behavioural, and emotional factors (Hatfield et al., 2007). Again, Hatfield and Rapson point out that passionate love is a complex emotion that includes feelings of jealousy as well as joy, sadness, and fear. Love in which passion is reciprocated has been found to be associated with satisfaction and happiness (Hatfield & Rapson, 1996). Examining studies on love and psychological well-being, it has been reported that there is a positive relationship between passionate love and finding meaning in one’s life (Yüksel, 2013). Ot has also been found that there is a strong and significant relationship between passionate love, being connected to each other in couples’ happiness (Büyükşahin & Hovardaoğlu, 2004), and that individuals in love have positive emotions and psychological well-being (Singh & Dhingra, 2014). A study using fMRI reported that individuals with high levels of passionate love and marital satisfaction had positive correlations in cortical and subcortical brain regions that support reward and motivation, goal-directed evaluation, empathy, emotion, and stress regulation (Acevedo et al., 2012).

When the above information from the literature is evaluated holistically, it can be seen that psychological well-being in romantic relationships is related to empathy, closeness, quality of sexual life, relationship stability and passionate love, and that studies have looked at one or more of these variables together, but there is no study that has looked at all of them together. This research was conducted to fill this gap in the field and to examine the predictive power of the variables of empathy, quality of sexual life, passionate love, relationship closeness, and relationship stability among women in romantic relationships according to the variable of psychological well-being. The second aim of the research is to examine the contribution of some demographic data (educational level, working life, and having children) to the psychological well-being of women in romantic relationships.

In line with this purpose, the research questions are listed below:

1. Are the variables of empathy, quality of sexual life, relationship stability, passionate love and closeness in romantic relationships significant predictors of the psychological well-being of women in romantic relationships?
2. Is there a significant relationship between the variable psychological well-being and the variables empathy, quality of sexual life, relationship stability, passionate love, and closeness in romantic relationships in women in romantic relationships?
3. Is there a significant difference between the psychological well-being of women in romantic relationships according to socio-demographic variables, in terms of empathy, quality of sexual life, relationship stability, passionate love and closeness in romantic relationships?

## 2. Materials and Methods

### 2.1. Research Model

The relational research model, one of the research models, was used in this study. The survey model is a research approach that aims to describe the past or current situation as it existed. The relational survey model is a research model that aims to determine the relationship between variables (Yöyen et al., 2024).

### 2.2. Sampling

A total of 415 women between the ages of 23 and 45, who had been in a romantic relationship for at least 12 months, participated in this study. The number of participants was determined by considering the number of people needed for different population sizes (Yazıcıoğlu & Erdoğan, 2004). The research form, distributed through the Google Forms application, was collected online using the snowball sampling method and on volunteerism. Online data collection occurred due to the COVID-19 quarantine measures taken during the study period. The snowball sampling method is used when researchers know very little about the population being studied (Etikan & Bala, 2017). In the snowball sampling method, one or more participants who met the inclusion and exclusion criteria of the study were randomly selected to recruit the additional participants. This continues until the desired number of participants is reached. The inclusion criteria for the sample were that participants were between 23 and 45 years old, in a romantic relationship for at least 12 months, and female. Exclusion criteria for the sample were defined as being outside the specified age range, having a romantic relationship of less than the specified duration, being male, having a psychiatric illness, having a psychiatric drug use, having a chronic physical illness and regular drug use related to it, and suffering from alcohol and drug abuse. The demographic information of the participants is presented in Table 1 and Table 2.

There were 415 participants in this study. A total of 5 (1.2%) participants had primary education, 9 (2.2%) participants had secondary education, 41 (9.9%) participants had high school education, 297 participants (71.6%) had a bachelor’s degree, 59 (14.2%) participants had a master’s degree, and 4 (1.0%) participants had a doctorate. Regarding relationship status, 188 (45.3%) participants were partners/lovers/dating, 46 (11.1%) participants were engaged/promised, and 181 (43.6%) participants were married. A total of 41 (9.9%) participants had been in a relationship for 12 months, 199 (48.0%) participants had been in a relationship for 12 months to 5 years, and 175 (42.2%) participants had been in a relationship for 5 years or more. A total of 300 (72.3%) participants had no children, 60 (14.5%) participants had one child, 55 (13.3%) participants had two or more children. A total of 232 (55.9%) participants worked and 183 (44.1%) did not work.

Table 2 shows the minimum, maximum, mean and standard deviation for age. The age range is 23–45, mean = 27.91, and standard deviation = 5.71.

### 2.3. Data Collection Instruments

The Sociodemographic Information Form, Psychological Well-Being Scale, Basic Empathy Scale, Sexual Quality of Life Scale, Passionate Love Scale, Romantic Relationship Closeness Scale, and Relationship Stability Scale developed by researchers were used in this study.

#### 2.3.1. Demographic Information Form

The prepared socio-demographic information form consists of five questions. There are questions about the participants’ age, level of education, relationship status (partner/lover/dating, engaged, and married), relationship duration (12 months, 12 months–5 years, 5 years and more), number of children, and employment status.

#### 2.3.2. Psychological Well-Being Scale (PWBS)

The Psychological Well-Being Scale is a self-reporting scale developed by Diener et al. (2009) to measure an individual’s current psychological well-being. The scale, which consists of 8 items, is answered between strongly disagree (1) and strongly agree (7). Scores range from 8 to 56. Individuals with high scores are considered to have high psychological resources. The Cronbach alpha internal consistency coefficient of the scale is 0.80 (Diener et al., 2009). When the responses of the sample in this study were evaluated, the result of the Cronbach alpha internal consistency coefficient of the scale was found to be 0.85.

#### 2.3.3. Basic Empathy Scale (BES)

This is a 20-item scale developed by Jolliffe and Farrington (2006), with 9 items measuring cognitive empathy and 11 items measuring emotional empathy. Responses are on a 5-point Likert scale ranging from ‘strongly disagree’ to ‘strongly agree’. Cognitive empathy is measured by items 3, 6, 9, 10, 12, 14, 16, 19, and 20, while emotional empathy is measured by items 1, 2, 4, 5, 7, 8, 11, 13, 15, 17, and 18. The lowest score that can be obtained from the scale for the cognitive empathy sub-dimension is 9 and the highest score is 45, while the lowest score that can be obtained for the emotional empathy sub-dimension is 11 and the highest score is 55. The Cronbach’s alpha coefficient values of the scale are 0.74 for the emotional dimension and 0.79 for the cognitive dimension (Jolliffe & Farrington, 2006). In this study, the Cronbach alpha internal consistency coefficient of the scale was found to be 0.78.

#### 2.3.4. Sexual Quality of Life Scale-Female (SQLS-F)

The Sexual Quality of Life Questionnaire was developed by Symonds et al. (2005). The scale is a 6-point Likert scale and consists of 18 items. These items are answered from strongly agree (1) to strongly disagree (6). Each item is to be answered by considering sexual life in the last four weeks. Before calculating the total score, the scores of items 1, 5, 9, 13, and 18 must be reversed. The range of scores that can be obtained from the scale is 18–108, and a high score indicates that the participant has a good quality of sexual life. The Cronbach alpha coefficient of the scale was reported to be 0.83 (Symonds et al., 2005). In this study, the Cronbach alpha internal consistency coefficient of the scale was found to be 0.95.

#### 2.3.5. Relationship Stability Scale (RSS)

The Relationship Stability Scale, developed by Rusbult et al. (1998), consists of 37 items and four sub-dimensions (relationship satisfaction, relationship investment, evaluation of quality of options, and commitment). The commitment sub-dimension consists of seven items and the other sub-dimensions consist of ten items. The items in the commitment dimension are Likert-type with a nine-point scale (1 = ‘strongly disagree’, 9 = ‘strongly agree’), while the first five items of the other sub-dimensions are Likert-type with a four-point scale (1 = ‘strongly disagree’, 4 = ‘strongly agree’); the last five items are Likert-type with a nine-point scale (1 = ‘strongly disagree’, 9 = ‘strongly agree’). In these sub-dimensions, the first five items are used to increase the measurement quality of the last five items. The Cronbach Alpha internal consistency coefficient of the scale is 0.90 for relationship satisfaction, 0.84 for evaluating the quality of alternatives, 0.84 for relationship investment, and 0.70 for the commitment dimension (Rusbult et al., 1998). In this study, the Cronbach Alpha internal consistency coefficient of the scale was determined to be 0.90 for the relationship satisfaction sub-dimension, 0.81 for the quality of alternatives sub-dimension, 0.82 for the relationship investment sub-dimension, and 0.83 for the commitment sub-dimension.

#### 2.3.6. Passionate Love Scale (PLS)

The Passionate Love Scale was developed by Hatfield and Sprecher (1986). The scale consists of 15 items and has a 9-point Likert scale. It is answered from not at all true (1) to definitely true (9). The highest score on the scale is 135 and the lowest is 15. The range of scores from 106 to 135 is interpreted as being madly, even recklessly, in love; the range from 86 to 105 is passionate but less intense feelings; the range from 66 to 85 is occasional outbursts of passion; the range from 45 to 65 is rarely passionate; and the range from 15 to 44 is the end of excitement (no passion at all). As the score on the scale increases, the level of passion increases, and conversely, as the score decreases, the level of passion decreases. The Cronbach alpha value of the scale is 0.89 (Hatfield & Sprecher, 1986). In this study, the Cronbach Alpha internal consistency coefficient of the scale was found to be 0.83.

#### 2.3.7. Romantic Relationship Closeness Scale (RRCS)

The Romantic Relationship Closeness Scale was developed by Ercan (2019). It consists of four sub-dimensions (self-disclosure, physical attraction, support, and trust) and 17 items, one of which is reverse-scored. The scale is a 4-point Likert-type scale (with options of never, sometimes, often, and always), and the lowest score that can be obtained from the entire scale is 17, and the highest score is 68. A high score indicates a close relationship, while a low score indicates a distant relationship. The Cronbach alpha internal consistency of the scale was reported to be 0.88 (Ercan, 2019). In this study, the Cronbach alpha internal consistency coefficient of the scale was found to be 0.87.

#### 2.3.8. Data Analysis

This study was approved by Social Sciences Research Ethics Board on 2 September 2021 with number 2021-66 and protocol number 2021-3/4. Data were collected online through the Google Forms application between October 2021 and May 2022 using the snowball sampling method on a voluntary basis. IBM SPSS 25 was used to analyse the data. Prior to basic analyses, data entries were checked, and missing data analysis was performed. The responses of 60 individuals who did not meet the age, gender, and minimum 12-month relationship criteria out of 475 individuals were excluded from this study. Frequencies and percentages were reported for the descriptive data analysis section and means and standard deviations for continuous variables. Normality assumptions for the study’s variables were assessed using box plots, stem and leaf plots, Q-Q plots, and skewness and kurtosis data. As defined by George and Mallery (2010), the distribution is considered normal if the kurtosis and skewness coefficients are between +2 and −2. Furthermore, if the coefficients of skewness and kurtosis are higher than +3 and −3, it indicates that the distribution is steeper and more skewed than normal (George & Mallery, 2010). Parametric tests were used in this study, as the data showed a normal and near-normal distribution. Pearson correlation analysis was used to examine the relationships between the scales, and multiple linear regression analysis and hierarchical regression analysis were performed for predictive value. To compare the scales used in the study with respect to socio-demographic variables, independent group *t*-test was used for variables with 2 categories and ANOVA for variables with more than 2 categories. In cases where the variances were not homogeneous, *t*-statistics and Welch statistics were used. The significance of the results was determined using a 95% confidence interval and a significance level of 0.05. Other levels are also reported.

## 3. Results

Table 3 shows the minimum and maximum scores, mean and standard deviation, skewness and kurtosis for the Basic Empathy Scale, Passionate Love Scale, Sexual Quality of Life Scale, Romantic Relationship Closeness Scale, Psychological Well-Being Scale, and Relationship Stability Scale.

It is observed that the scores of the Romantic Relationships Closeness Scale (F(2–99.540) = 3.482; *p* < 0.05) and the sub-dimensions of the Relationship Stability Scale Quality of Choice Evaluation (F(2–412) = 4.460; *p* < 0.05) and Relationship Investment (F(2–412) = 3.742; *p* < 0.01) differ significantly according to educational status. It was observed that participants with an associate/undergraduate and graduate level of education had higher scores on the Evaluation of Quality of Choice Evaluation sub-dimension of the Relationship Stability Scale than participants with only primary/secondary/high school education. It was observed that participants with primary/secondary/high school education had higher scores on the Relationship Investment sub-dimension of the Relationship Stability Scale than participants with postgraduate education. Although the test results were significant for scores on the Romantic Relationships Closeness Scale, the results of the paired comparisons test were not significant. It was found that the scores for the relationship satisfaction and attachment sub-dimensions of the Basic Empathy Scale, Sexual Quality of Life Scale, Passionate Love Scale, Psychological Well-Being Scale, and Relationship Stability Scale did not show statistically significant differences according to educational status (*p* > 0.05). The results are presented in Table 4.

Sexual Quality of Life Scale (F(2–412) = 3.927; *p* < 0.05). Romantic Relationships Closeness Scale (F(2–142.204) = 3.372; *p* < 0.01). It was observed that the scores of the sub-dimensions of the Psychological Well-Being Scale (F(2–412) = 7.857; *p* < 0.01) and the Relationship Stability Scale Assessing the Quality of Choice Evaluation (F(2–412) = 3.693; *p* < 0.05) and Attachment (F(2–214.158) = 14.658; *p* < 0.01) differed significantly according to relationship status. It was observed that the scores of the Sexual Quality of Life Scale and the Psychological Well-Being Scale of the married participants were higher than the scores of the partner/lover/dating participants. It was observed that the scores of the Romantic Relationships Closeness Scale and the Assessment of Quality of Choice Evaluation sub-dimensions of the partner/lover/dating participants were higher than the scores of the married/engaged participants. It was observed that the Attachment sub-dimension scores of the engaged/promised participants were higher than those of the married and partner/lover/dating participants, according to the Basic Empathy Scale. It was found that the scores of the Passionate Love Scale and the sub-dimensions of the Relationship Stability Scale, Relationship Satisfaction, and Relationship Investment did not show a statistically significant difference according to relationship status (*p* > 0.05). The results are presented in Table 5.

It is observed that the scores of the sub-dimensions of the Psychological Well-Being Scale (F(2–412) = 4.872; *p* < 0.01) and the Relationship Stability Scale Relationship Investment (F(2–412) = 10.111; *p* < 0.01) differ significantly according to the duration of the relationship. It was observed that the Psychological Well-Being Scale scores of participants who had been in a relationship for 5 years or more were higher than those of participants who had been in a relationship for 12 months to 5 years. Participants who had been in a relationship for 5 years or more had higher scores on the Relationship Investment subscale than participants who had been in a relationship for less than 12 months and between 12 months and 5 years. The results are presented in Table 6.

Romantic relationship closeness scale (F(2–90.631) = 7.825; *p* < 0.01), Psychological Well-being Scale (F(2–412) = 6.603; *p* < 0.01), Relationship Stability Scale Quality of Choice Evaluation (F(2–412) = 4.421; *p* < 0.05) sub-dimension scores are significantly different according to the number of children. Participants with no children were found to have higher scores on the Romantic Relationship Closeness Scale than participants with one child and two or more children. Participants with one child and two or more children were found to have higher scores on the Psychological Well-being Scale than participants without children. Participants with no children were found to have higher scores on the Quality of Choice Evaluation sub-dimension than participants with two or more children. The results are presented in Table 7.

It is observed that the scores of the Relationship Stability Scale Assessment of the Quality of Choice Evaluation sub-dimension (*t*(412,501) = −2.261; *p* < 0.05) and the Passionate Love Scale (*t*(413) = 4.279; *p* < 0.01) differ significantly according to employment status. It was observed that the Passionate Love Scale scores of unemployed participants were higher than those of employed participants. It was observed that the scores of the employed participants on the sub-dimension Quality of Choice Evaluation were higher than those of the unemployed participants. The results are presented in Table 8.

Table 9 shows the results of the Pearson correlation analysis examining the relationships between scores on the Basic Empathy Scale, Passionate Love Scale, Sexual Quality of Life Scale, Romantic Relationship Closeness Scale, Psychological Well-Being Scale, and Relationship Stability Scale. A statistically significant relationship was found between scores on the Basic Empathy Scale and the Passionate Love Scale (r = 0.205; *p* < 0.01). A positive weak level between the Psychological Well-Being Scale (r = 0.101; *p* < 0.05). A positive weak level between the RSS relationship investment (r = 0.164; *p* < 0.01) and RSS attachment (r = 0.136; *p* < 0.01) scores. It was found that there was a positive weak level relationship between Passionate Love Scale scores and Sexual Quality of Life Scale (r = 0.133; *p* < 0.01), a positive moderate level relationship between Romantic Relationship Closeness Scale (r = 0.507; *p* < 0.01), a positive moderate level relationship between RSSS Relationship Satisfaction (r = 0.318; *p* < 0.01), a negative weak level relationship between RSS Relationship Investment (r = 0.494; *p* < 0.01) and a positive moderate level relationship between RSSS-Attachment (r = 0.594; *p* < 0.01) scores. It was found that there was a statistically significant positive weak level relationship between the Sexual Quality of Life Scale scores and the Romantic Relationship Closeness Scale (r = 0.288; *p* < 0.01), a positive moderate level relationship between the Psychological Well-Being Scale (r = 0.308; *p* < 0.01), a positive moderate level relationship between RSS Relationship Satisfaction (r = 0.342; *p* < 0.01), a negative weak level relationship between RSS Relationship Investment (r = −0.124; *p* < 0.01) and a positive weak level relationship between RSS Attachment (r = 0.207; *p* < 0.01) scores. It was found that there was a positive moderate correlation between scores on the Romantic Relationship Closeness Scale (r = 0.263; *p* < 0.01) and the Psychological Well-Being Scale (r = 0.308; *p* < 0.01), a positive moderate correlation between scores on the IRQ Relationship Satisfaction (r = 0.561; *p* < 0.01), a negative weak correlation between scores on the IRQ Quality of Choice (r = −0.180; *p* < 0.01), and a positive moderate correlation between scores on the IRQ Attachment (r = 0.488; *p* < 0.01). It was found that there was a positive moderate correlation between scores on the Psychological Well-Being Scale and Relationship Satisfaction (r = 0.394; *p* < 0.01) and a positive weak correlation between scores on the IRQ-Attachment (r = 0.194; *p* < 0.01).

To determine the predictive effect of scores on the Basic Empathy Scale, Passionate Love Scale, Sexual Quality of Life Scale, Romantic Relationships Closeness Scale, and Relationship Stability Scale on scores on the Psychological Well-Being Scale, the stepwise method was used. The established model was found to be statistically significant (F(3.411) = 33.654. *p* < 0.01). The model explains 19.6% of the variance in the Psychological Well-Being Scale scores. Relationship satisfaction (β = 0.324. *t* = 6.882. *p* < 0.01), sexual life quality (β = 0.197. *t* = 4.188. *p* < 0.01) and empathy (β = 0.088. *t* = 1.997. *p* < 0.05) scores were found to be statistically significant predictors of Psychological Well-Being Scale scores. Other scale scores were not found to be statistically significant predictors. The results are presented in Table 10.

To determine the predictive effect of Psychological Well-Being Scale scores on Basic Empathy Scale, Passionate Love Scale, Sexual Quality of Life Scale, Romantic Relationships Closeness Scale and Relationship Stability Scale scores, some demographic variables were included in the model and hierarchical regression method was used. In the first step, empathy, sexual life quality and relationship satisfaction were included in the model. The model explains 19.7% of the variance in the Psychological Well-Being Scale scores. It was found that relationship satisfaction (β = 0.324. *t* = 6.882. *p* < 0.01), quality of sexual life (β = 0.197. *t* = 4.188. *p* < 0.01), and empathy (β = 0.088. *t* = 1.997. *p* < 0.05) scores were statistically significant predictors of Psychological Well-Being Scale scores. In a second step, the variables relationship status, relationship duration and number of children were included in the model. It was found that the number of children variable (β = 0.153. *t* = 2.833. *p* < 0.01) predicted Psychological Well-Being Scale scores. Relationship status and relationship duration were not found to be significant predictors. The final model was found to explain 29.2% of the variance in Psychological Well-Being Scale scores. The results are presented in Table 11.

## 4. Discussion

In this study, which investigated the predictive and contributing variables in the psychological well-being of women in romantic relationships, the research findings can be summarised as follows: in the psychological well-being of women in romantic relationships, empathy, sexual life quality, relationship satisfaction and having children are the predictive variables, respectively. The results of the contributing variables are as follows: in women in romantic relationships, as the level of education increases, the dimension of evaluating options in the relationship increases, and as the level of education decreases, the dimension of investing in the relationship increases. The quality of sexual life and psychological well-being is higher among married women than among unmarried women. Intimacy and the assessment of the quality of the relationship in a romantic relationship are higher among unmarried women than among married women. With increasing duration of the romantic relationship, psychological well-being, and investment in the relationship increase. For women in romantic relationships, having children increases psychological well-being, while not having children increases intimacy and evaluation of the quality of the relationship in a romantic relationship. Passionate love is higher among unemployed women, and evaluation of relationship quality is higher among employed women.

The findings of the study that relationship satisfaction, empathy, quality of sexual life, and having children predict psychological well-being, and that psychological well-being is higher among those who are married, in long-term relationships and have children, are supported by the literature. Studies have reported that married people have higher psychological well-being than those who do not have a marital status (Grundström et al., 2021; Kim & Mckenry, 2002). Marriage provides the social support resources that individuals need (Lansford et al., 2024), providing meaning and emotional support in life (Kim & Mckenry, 2002), and reduces vulnerability to mental health conditions. As with marriage, the length of the relationship (5 years or more) also contributes to psychological well-being. Studies have found higher levels of psychological well-being in individuals who have been together for more than 60 months compared to those who have been together for 0–12 months (Doyle & Molix, 2014). The long duration of the relationship may be an indicator of the harmony, satisfaction, and quality of the relationship. At the same time, it may contribute to psychological well-being by satisfying the psychological and social needs of individuals such as trust, value, protection, and solidarity with the partner, being able to look to the future with confidence, participating in society, being proud of each other, not being alone, and a sense of partnership in terms of the possibility of a future in the relationship.

The predictive value of empathy for psychological well-being is supported by many studies in the literature (Bourgault et al., 2015; Carnicer & Calderon, 2014; Khajeh et al., 2014; O’Conner et al., 2015; Vinayak & Judge, 2018). One of the most important factors in ensuring psychological well-being is the establishment of positive relationships with others. An empathic individual increases their positive perception not only of their partner, but also of their own through their empathic feelings and actions: an empathic person who can recognise their own feelings can also understand the emotional state and intentions of others. This situation can create good feelings in the person. In order to talk about the level of psychological well-being of a person, they must have the characteristics of being sincere, trusting their interpersonal relationships, having the ability to empathise, being able to establish closeness, being able to help, being able to make decisions according to their own thoughts and actions, being able to control themselves, being able to choose environments that are suitable for them, developing their potential, trying to understand the purpose of life, being aware of their feelings, behaviours and motivations and having a positive attitude towards them. Therefore, the fact that psychological well-being is similar to the concepts mentioned in its own definition explains empathy. Again, by definition, empathy requires the desire and ability to understand and care about the pain of another person, a loving attitude. In this context, empathy is both a precursor to love and an important component or basic requirement of love. Therefore, it can be assumed that in the presence of empathy, couples are more likely to continue their relationship and their commitment to their partner and satisfaction in their relationship increases.

There are conflicting findings in the literature on the role of childbearing on psychological well-being. Some studies report that the number of children has no effect on women’s psychological well-being, while others report that the average psychological well-being of participants with two children is higher than those with three or more children (Maharramova & Kumcağız, 2021). From an evolutionary perspective, individuals choose a mate to ensure the continuity of the species. Individuals enter into a romantic relationship in order to choose a partner, reproduce with them, and raise the child (Buss, 1988). In this respect, the fact that a woman has a child after a romantic relationship is consistent with the evolutionary goal being achieved. This value of having children in the literature can be explained by the fact that the effects of psychological well-being, which is multidimensional, vary from person to person. There is information in the literature that the relationship between having children and psychological well-being is sometimes high in couples who do not have children, and sometimes having children above a certain number negatively affects psychological well-being levels due to increased anxiety about responsibility and burden (Umberson et al., 2010). An increase in the number of children can lead to an increase in responsibilities and daily problems in marriage. This can have a negative impact on psychological well-being. However, the quality of the relationship between parents and their children can also contribute to their mental health and psychological well-being. Therefore, parenthood is not a single experience that affects psychological well-being, and not having children does not have the same outcome for everyone.

Another predictor of psychological well-being in this study was the quality of sexual life. This finding is consistent with the literature (Holmberg et al., 2010; Stackert & Bursik, 2003). Other studies that support the results of this study include the information that people who have a regular, healthy and satisfying sexual life have healthier romantic relationships and higher psychological well-being (Biss & Horne, 2005), that high sexual dissatisfaction in women negatively affects their psychological well-being (Davison et al., 2009), and that the well-being of the person and their partner is one of the necessary motivations for initiating sexual intercourse (Basson, 2005). Although the finding that the quality of sexual life of married women in this study was higher than that of women in other relationship statuses (engaged, dating, partner, etc.) seems to contradict the literature, which indicates that the highest sexual satisfaction is found among engaged individuals rather than married ones (Birnie-Porter & Hunt, 2015), it is an understandable result within the socio-cultural context of the country in which the study was conducted. As the culture in which the study was conducted has a traditional social structure, sexual intercourse is only associated with having children and being married. In the cultural structure of the country, honour is generally seen as sexual purity. Female sexuality is strictly controlled, and honour killings occur if this requirement is not met.

Another finding of this study is that relationship satisfaction plays a role as a predictor of psychological well-being. One dimension of psychological well-being is an individual’s self-acceptance. Self-acceptance allows the individual to develop positive relationships with others, to be autonomous, to have the ability to manage their environment, to have a purpose in life, and to have continuous personal development. This can help the individual to develop positive relationships with others. There is evidence on this in the literature. Towler’s research concluded that the factors that lead to successful patterns of interaction with women’s partners are related to well-being (Towler & Stuhlmacher, 2013). Another study reported that levels of psychological well-being are related to relationship satisfaction and specific dimensions of relationship quality (conflict, closeness, and commitment), and that low levels of psychological distress in one partner negatively affect relationship satisfaction for both partners (Otis et al., 2006). There are also studies that report that continuing an unhappy marriage is associated with lower levels of happiness, life satisfaction, general health, and psychological well-being than staying married forever (Hawkins & Booth, 2005). When a relationship is not satisfying, the health of the individual is negatively affected. Healthier individuals are more likely to marry and stay married and/or have more financial resources and social support than those who are not married, while having less stress and risky health habits. In short, the relationship between relationship satisfaction and psychological well-being is bidirectional (Braithwaite & Holt-Lunstad, 2017). While relationship satisfaction can be a predictor of psychological well-being, the opposite can also be the case: relationship satisfaction can predict psychological well-being.

The results of the research show that the dimension of assessing the quality of options in relationship stability increases with the level of education (among women with a bachelor’s or master’s degree compared to women with only a primary, secondary, or high school education), in cases where the relationship status is not officially binding (among women who are partners, dating, or lovers compared to married women), in cases where there are no children and among working women. The research findings are in line with the literature. It is reported in the literature that people’s scores for evaluating the quality of options decrease depending on the number of children and that the loss of freedom and reduced autonomy experienced with the birth of a child requires a great deal of adjustment for women and brings with it some difficulties (Twenge et al., 2003), and as the level of education and participation in working life increases, so do the scores for assessing the quality of options (Büyükşahin, 2006).

The socio-demographic results of this study show that the sub-dimension scores for assessing the quality of options in relationships are higher among women who are employed, have no children and have a bachelor’s degree than among participants who are not employed, have children and have a primary/secondary/high school education. These findings also need to be evaluated in a cultural context. The fact that there is a high level of traditional and low level of egalitarian perception of gender roles in the country where the study was conducted affects the psychological well-being of individuals through their gender roles. Women’s roles, attitudes and behaviours in the country’s cultural perception of gender are based on values such as compassion, mercy, protection of the weak, kindness, sensitivity, understanding and relationship building. These values lead women to behave in ways such as marrying at an early age, becoming mothers at a young age, spending time at home organising the health and well-being of their spouses and children, and investing in their romantic relationships. In other words, the role of mother and wife, which is one of the gender roles attributed to women, is effective in keeping women in their relationships. However, there are also women in the same culture who go beyond the traditional gender role. These women can achieve higher education and economic status. In this status, money and the “sense of freedom and individuality” that comes with a professional career can protect women’s psychological well-being from the negative effects of moving away from traditional gender roles.

The research results show that closeness in romantic relationships is higher among those who are partners, lovers and are dating than among those who are married, and among women who do not have children than among those who do. The fact that women who do not have children have higher romantic intimacy scores than women who have children can be interpreted as women who have children focusing on their children and reducing their romantic intimacy with their spouse. This is because parenthood is thought to involve sacrifices, losses, and missed opportunities in professional, social, and emotional life. There are similar studies in the literature (Holland et al., 2016). It has been reported that closeness in romantic relationships varies according to relationship status, that low levels of closeness are found in non-serious relationships (casual dating, short-term non-serious relationships, etc.) compared to more serious relationship statuses (exclusive dating, engaged, married, etc.), and that the highest levels of romantic closeness are found in engaged individuals (Birnie-Porter & Hunt, 2015).

The finding of this study that the commitment sub-dimension scores of the engaged participants were higher than those of the married and partner/lover/dating participants is significant in terms of the theory of love triangles, proposed by Sternberg. Sternberg predicted that commitment is a gradually increasing situation, accelerating as the relationship becomes stronger and eventually levelling off (Sternberg, 1986). Therefore, the finding that commitment is highest in engaged couples and lowest in dating relationships supports Sternberg’s theory. Studies have found that there is an increase in closeness, passion, and commitment from casual relationships to serious and engaged couples (Gao, 2001). According to Sternberg, a sense of closeness and commitment is evident in a loving relationship (Sternberg, 1986). According to Lewis, in order for a couple to seek satisfaction in their relationship, they need to cope with commitment, power, and closeness (Levin, 2007).

The research results show that passionate love scores are higher for employed women than for unemployed women. There is no information in the literature to support this finding. However, the fact that working women also score higher on the quality of options for relationship stability may mean that the social, psychological, and economic resources available to the woman as a result of her working life are more conducive to starting and maintaining a relationship with her partner. This comfort may ensure that the partner chosen is emotional rather than a logical choice. In addition, women may turn to passionate love in order to experience the passion, closeness, commitment, trust, respect, and affection that love brings, and to avoid the professional and social burdens of working life.

## 5. Conclusions

This study examined the predictors of psychological well-being in women in romantic relationships. It was concluded that relationship satisfaction, quality of sexual life, empathy, and having children predicted psychological well-being. It was also concluded that psychological well-being was higher in married people and those who had been in a romantic relationship for a long time. In addition to the above information, this study found that higher levels of education, employment, not having children, and not being in a marital relationship increased a woman’s tendency to evaluate options in their romantic relationships. As the level of education decreases, investment in the relationship increases, while increasing the seriousness of the relationship (from partner, dating relationship to marriage) increases the quality of sexual life and the level of commitment in the relationship. However, increasing the seriousness of the relationship decreases the closeness in romantic relationships.

### Recommendations

It has been observed that while studies of psychological well-being in romantic relationships tend to focus more on married or divorced people, there are few studies that look at all relationship situations together. It is, therefore, felt that the field would be enriched by addressing the lack of research in this area. In addition, there is limited research on intimacy and passionate love in romantic relationships. Increasing the number of studies on these topics will contribute to the field.

People over the age of 45 were not included in this study so that the effect of menopause would not be a confounding variable. It is expected that a separate study will be conducted for the sample of women over the age of 45. This will add to the body of knowledge by providing comprehensive results for this sample.

It was concluded that women’s relationship investment, commitment, satisfaction, and quality of choice ratings are effective in maintaining their relationships, but only relationship satisfaction predicts psychological well-being. Therefore, couples’ workshops and psychoeducation for relationship satisfaction and counselling studies that include other elements of the relationship (investment, commitment) may contribute to the maintenance of women’s psychological well-being.

The fact that the quality of women’s sexual life is a predictor of psychological well-being shows the importance of sexual life. Therefore, women who are sexually active/inactive should be informed and educated on the subject by educational institutions, family health centres, and the media. All this will help women to have a quality sexual life and contribute to their psychological well-being.

It has been found that the psychological well-being of women in romantic relationships varies according to the number of children and the duration of the relationship. This finding can be used to reach out to women’s communities and provide support for private life planning.

Psychological well-being, empathy, sexuality, relationship stability, love, and intimacy in romantic relationships are related to women’s mental health. Therefore, it is expected that all the findings will contribute to the work of expert researchers in fields such as counselling psychology, family sociology, social psychology, and couples’ therapy.

This is a quantitative study. In the future, researchers can conduct qualitative research with women in romantic relationships to test the effect of the same study.

In addition, a limitation of this study is related to the research method and sampling. The snowball sampling method was used. The sample is restricted to Turkey. In this context, the generalisability of the results is limited. It is recommended that future studies conduct randomised and cross-cultural studies. This may increase the generalisability and comparability of the data obtained.

## Figures and Tables

**Table 1:** Demographic characteristics of participants.

Variable	Groups	n	f (%)
Educational Status	Primary School	5	1.2
	Secondary School	9	2.2
	High School	41	9.9
	Bachelor’s Degree	297	71.6
	Master’s Degree	59	14.2
	Doctorate	4	1.0
Relationship Status	Partner/Lover/Dating	188	45.3
	Engaged	46	11.1
	Married	181	43.6
Relationship Duration	12 Months	41	9.9
	12 Months–5 Years	199	48.0
	Over 5 Years	175	42.2
Number of Children	No Child	300	72.3
	One Child	60	14.5
	2+ Children	55	13.3
Working Status	Employed	232	55.9
	Unemployed	183	44.1

**Table 2:** Descriptive Statistics for Age.

Scales	Min.	Max.	$\bar{x}$	sd
Age	23	45	27.91	5.71

**Table 3:** Descriptive statistics for the Basic Empathy Scale, Passionate Love Scale, Sexual Quality of Life Scale, Romantic Relationship Closeness Scale, Psychological Well-Being Scale, and Relationship Stability Scale.

Scales	Min.	Max.	$\bar{x}$	sd	Skewness	Kurtosis
Basic Empathy Scale	48	88	76.59	6.17	−0.806	1.790
Passionate Love Scale	47	135	111.77	15.29	−1.187	1.875
Sexual Quality of Life Scale	0	100	78.51	22.65	−1.385	1.244
Romantic Relationships Closeness Scale	32	64	55.47	5.99	−1.345	1.520
Psychological Well-Being Scale	8	56	44.03	7.85	−0.887	1.333
RSS Relationship Satisfaction	15	45	38.97	6.76	−1.431	1.582
RSS Quality of Choice Evaluation	5	45	18.82	10.36	0.414	−0.716
RSS Relationship Investment	5	45	26.79	10.44	−0.094	−0.929
RSS Attachment	33	63	58.01	7.47	−1.768	2.172

**Table 4:** ANOVA results for the analysis of the scores of the Basic Empathy Scale, Passionate Love Scale, Sexual Quality of Life Scale, Romantic Relationships Closeness Scale, Psychological Well-Being Scale and Relationship Stability Scale according to educational status.

Scales	Groups	n	$\bar{x}$	sd	F	sd	*p*	Difference
Basic Empathy Scale	Primary–Secondary School/High School	55	75.89	4.52	0.948 ^w^	2 110.168	0.391	—
	Associate Degree/Undergraduate	297	76.81	6.21				
	Postgraduate	63	76.14	7.13				
Passionate Love Scale	Primary–Secondary School/High School	55	110.36	17.97	1.269 ^w^	2 93.123	0.286	—
	Associate Degree/Undergraduate	297	112.60	13.88				
	Postgraduate	63	109.08	18.63				
Sexual Quality of Life Scale	Primary–Secondary School/High School	55	79.21	20.96	0.056	2 412	0.946	—
	Associate Degree/Undergraduate	297	78.28	23.04				
	Postgraduate	63	78.99	22.56				
Romantic Relationships Closeness Scale	Primary–Secondary School/High School	55	52.82	8.34	3.482 ^w^	2 99.540	0.035 *	
	Associate Degree/Undergraduate	297	55.86	5.58				
	Postgraduate	63	55.98	4.87				
Psychological Well-Being Scale	Primary–Secondary School/High School	55	44.85	7.77	0.451	2 412	0.637	—
	Associate Degree/Undergraduate	297	43.81	8.12				
	Postgraduate	63	44.30	6.62				
RSS Relationship Satisfaction	Primary–Secondary School/High School	55	37.85	7.75	0.700 ^w^	2 108.782	0.499	—
	Associate Degree/Undergraduate	297	39.12	6.88				
	Postgraduate	63	39.22	5.04				
RSS Quality of Choice Evaluation	Primary–Secondary School/High School	55	15.05	10.07	4.460	2 412	0.012 *	2 > 1
	Associate Degree/Undergraduate	297	19.24	10.31				3 > 1
	Postgraduate	63	20.14	10.28				
RSS Relationship Investment	Primary–Secondary School/High School	55	28.91	10.97	3.742	2 412	0.025 *	1 > 3
	Associate Degree/Undergraduate	297	27.02	9.97				
	Postgraduate	63	23.86	11.63				
RSS Attachment	Primary–Secondary School/High School	55	56.58	9.34	1.251 ^w^	2 97.069	0.291	—
	Associate Degree/Undergraduate	297	58.40	7.08				
	Postgraduate	63	57.41	7.41				

* *p* < 0.05; w: Welch test.

**Table 5:** ANOVA results for the analysis of the scores of the Basic Empathy Scale, Passionate Love Scale, Sexual Quality of Life Scale, Romantic Relationships Closeness Scale, Psychological Well-Being Scale and Relationship Stability Scale according to relationship status.

Scales	Groups	n	$\bar{x}$	sd	F	sd	*p*	Difference
Basic Empathy Scale	Partner/Lover/Dating	188	76.43	6.19	1.919	2 412	0.148	—
	Engaged	46	78.26	6.33				
	Married	181	76.33	6.06				
Passionate Love Scale	Partner/Lover/Dating	188	111.52	15.07	0.928	2 412	0.396	-
	Engaged	46	114.65	10.95				
	Married	181	111.30	16.41				
Sexual Quality of Life Scale	Partner/Lover/Dating	188	75.18	23.77	3.927	2 412	0.020 *	3 > 1
	Engaged	46	79.57	19.72				
	Married	181	81.70	21.77				
Romantic Relationships Closeness Scale	Partner/Lover/Dating	188	56.14	5.18	3.372 ^w^	2 142.204	0.037 *	1 > 3
	Engaged	46	56.26	4.33				
	Married	181	54.58	6.98				
Psychological Well-Being Scale	Partner/Lover/Dating	188	42.56	7.99	7.857	2 412	0.000 **	3 > 1
	Engaged	46	43.39	6.65				
	Married	181	45.71	7.70				
RSS Relationship Satisfaction	Partner/Lover/Dating	188	38.67	6.09	2.502 ^w^	2 135.647	0.086	—
	Engaged	46	40.72	5.55				
	Married	181	38.83	7.61				
RSS Quality of Choice Evaluation	Partner/Lover/Dating	188	20.27	10.17	3.693	2 412	0.026 *	1 > 3
	Engaged	46	18.70	8.79				
	Married	181	17.35	10.77				
RSS Relationship Investment	Partner/Lover/Dating	188	25.97	10.31	2.026	2 412	0.133	—
	Engaged	46	25.59	11.19				
	Married	181	27.96	10.32				
RSS Attachment	Partner/Lover/Dating	188	57.49	7.77	14.658 ^w^	2 214.158	0.000 **	2 > 1
	Engaged	46	60.91	3.04				2 > 3
	Married	181	57.81	7.81				

* *p* < 0.05; ** *p* < 0.01; w: Welch test.

**Table 6:** ANOVA results for the analysis of the scores of the Basic Empathy Scale, Passionate Love Scale, Sexual Quality of Life Scale, Romantic Relationship Closeness Scale, Psychological Well-Being Scale and Relationship Stability Scale according to the relationship duration.

Scales	Groups	n	$\bar{x}$	sd	F	sd	*p*	Difference
Basic Empathy Scale	12 Months	41	76.90	5.20	0.227	2 412	0.797	—
	12 Months–5 Years	199	76.38	6.11				
	Over 5 Years	175	76.75	6.46				
Passionate Love Scale	12 Months	41	108.37	17.81	1.171	2 412	0.311	—
	12 Months–5 Years	199	111.93	13.66				
	Over 5 Years	175	112.39	16.36				
Sexual Quality of Life Scale	12 Months	41	82.66	20.57	1.200	2 412	0.302	—
	12 Months–5 Years	199	77.03	23.01				
	Over 5 Years	175	79.23	22.67				
Romantic Relationships Closeness Scale	12 Months	41	55.05	6.09	1.751 ^w^	2 109.256	0.178	—
	12 Months–5 Years	199	56.04	5.05				
	Over 5 Years	175	54.93	6.87				
Psychological Well-Being Scale	12 Months	41	43.05	10.10	4.872	2 412	0.008 **	3 > 2
	12 Months–5 Years	199	43.00	7.70				
	Over 5 Years	175	45.42	7.24				
RSS Relationship Satisfaction	12 Months	41	40.17	5.05	1.083	2 412	0.340	—
	12 Months–5 Years	199	38.56	6.83				
	Over 5 Years	175	39.15	7.01				
RSS Quality of Choice Evaluation	12 Months	41	17.68	10.16	0.712	2 412	0.491	—
	12 Months–5 Years	199	19.42	10.06				
	Over 5 Years	175	18.41	10.76				
RSS Relationship Investment	12 Months	41	23.44	9.09	10.111	2 412	0.000 **	3 > 1
	12 Months–5 Years	199	25.22	10.61				3 > 2
	Over 5 Years	175	29.37	10.01				
RSS Attachment	12 Months	41	57.88	7.09	0.704	2 412	0.704	—
	12 Months-5 Years	199	57.59	7.79				
	Over 5 Years	175	58.51	7.21				

** *p* < 0.01; w: Welch test.

**Table 7:** ANOVA results for the analysis of the scores of the Basic Empathy Scale, Passionate Love Scale, Sexual Quality of Life Scale, Romantic Relationship Closeness Scale, Psychological Well-Being Scale and Relationship Stability Scale according to the number of children.

Scales	Groups	n	$\bar{x}$	sd	F	sd	*p*	Difference
Basic Empathy Scale	No Child	300	76.71	6.10	0.379	2 412	0.685	—
	One Child	60	75.95	6.36				
	2+ Children	55	76.65	6.37				
Passionate Love Scale	No Child	300	112.44	14.37	2.428 ^w^	2 97.999	0.094	—
	One Child	60	113.42	12.06				
	2+ Children	55	106.31	21.31				
Sexual Quality of Life Scale	No Child	300	77.63	23.04	1.824	2 412	0.163	—
	One Child	60	83.67	20.28				
	2+ Children	55	77.72	22.62				
Romantic Relationships Closeness Scale	No Child	300	56.13	5.15	7.825 ^w^	2 90.631	0.001 **	1 > 2
	One Child	60	55.95	5.68				1 > 3
	2+ Children	55	51.38	8.59				
Psychological Well-Being Scale	No Child	300	43.17	7.64	6.603	2 412	0.002 **	2 > 1
	One Child	60	46.05	8.78				3 > 1
	2+ Children	55	46.47	7.13				
RSS Relationship Satisfaction	No Child	300	39.36	5.98	1.509 ^w^	2 88.760	0.227	—
	One Child	60	38.50	8.11				
	2+ Children	55	37.36	8.74				
RSS Quality of Choice Evaluation	No Child	300	19.72	10.15	4.241	2 412	0.015 *	1 > 3
	One Child	60	16.83	10.26				
	2+ Children	55	16.07	11.03				
RSS Relationship Investment	No Child	300	26.11	10.61	2.463	2 412	0.086	—
	One Child	60	28.05	9.35				
	2+ Children	55	29.13	10.36				
RSS Attachment	No Child	300	58.06	7.38	2.157 ^w^	2 101.916	0.121	—
	One Child	60	59.27	5.85				
	2+ Children	55	56.35	9.19				

* *p* < 0.05; ** *p* < 0.01; w: Welch test.

**Table 8:** Independent groups *t*-test results for the analysis of the scores of the Basic Empathy Scale, Passionate Love Scale, Sexual Quality of Life Scale, Romantic Relationships Closeness Scale, Psychological Well-Being Scale and Relationship Stability Scale according to employment status.

Scales	Groups	n	$\bar{x}$	sd	t	sd	*p*
Basic Empathy Scale	EEmployed	232	76.50	6.19	−0.352	413	0.725
	Unemployed	183	76.71	6.16			
Passionate Love Scale	Employed	232	110.31	16.48	−2.261	412.501	0.024 *
	Unemployed	183	113.63	13.45			
Sexual Quality of Life Scale	Employed	232	78.17	22.98	−0.345	413	0.730
	Unemployed	183	78.94	22.29			
Romantic Relationships Closeness Scale	Employed	232	55.29	6.04	−0.702	413	0.483
	Unemployed	183	55.70	5.95			
Psychological Well-Being Scale	Employed	232	44.58	7.98	1.625	413	0.105
	Unemployed	183	43.32	7.65			
RSS Relationship Satisfaction	Employed	232	38.94	6.70	−0.084	413	0.933
	Unemployed	183	39.00	6.84			
RSS Quality of Choice Evaluation	Employed	232	20.72	10.53	4.279	413	0.000 **
	Unemployed	183	16.42	9.65			
RSS Relationship Investment	Employed	232	26.85	10.81	0.133	413	0.894
	Unemployed	183	26.72	9.98			
RSS Attachment	Employed	232	57.80	7.61	−0.630	413	0.529
	Unemployed	183	58.27	7.32			

* *p* < 0.05; ** *p* < 0.01; a statistics used when variances are not homogeneous.

**Table 9:** Pearson correlation analysis results for examining the relationships between Basic Empathy Scale, Passionate Love Scale, Sexual Quality of Life Scale, Romantic Relationship Closeness Scale, Psychological Well-Being Scale and Relationship Stability Scale Scores.

Scales	1	2	3	4	5	6	7	8	9
Basic Empathy Scale	1								
Passionate Love Scale	0.205 **	1							
Sexual Quality of Life Scale	0.011	0.133 **	1						
Romantic Relationships Closeness Scale	0.080	0.507 **	0.288 **	1					
Psychological Well-Being Scale	0.101 *	0.088	0.308 **	0.263 **	1				
RSS Relationship Satisfaction	0.032	0.318 **	0.342 **	0.561 **	0.394 **	1			
RSS Quality of Choice Evaluation	−0.017	−0.208 **	−0.067	−0.180 **	−0.022	−0.240 **	1		
RSS Relationship Investment	0.164 **	0.494 **	−0.124 *	0.078	−0.054	−0.020	−0.114 *	1	
RSS Attachment	0.136 **	0.594 **	0.207 **	0.488 **	0.194 **	0.510 **	−0.333 **	0.258 **	1

* *p* < 0.05; ** *p* < 0.01.

**Table 10:** Results of multiple linear regression analysis on the predictive effect of Psychological Well-Being Scale Scores on Basic Empathy Scale, Passionate Love Scale, Sexual Quality of Life Scale, Romantic Relationship Closeness Scale and Relationship Stability Scale scores.

Dependent Variable	Independent Variable	B	Standard Error_B_	Beta	*t*	*p*
Psychological Well-Being Scale	Fixed	15.384	4.743		3.244	0.001
	Relationship Satisfaction	0.376	0.055	0.324	6.882	0.000 **
	Quality of Sexual Life	0.068	0.016	0.197	4.188	0.000 **
	Empathy	0.112	0.056	0.088	1.997	0.046 *
R = 0.444 R^2^ = 0.197 Sd: 3/411 F: 33.654 *p* = 0.000 **						

** *p* < 0.01. * *p* < 0.05.

**Table 11:** Hierarchical regression analysis results regarding the predictive effect of Psychological Well-Being Scale Scores on Basic Empathy Scale, Passionate Love Scale, Sexual Quality of Life Scale, Romantic Relationship Closeness Scale and Relationship Stability Scale Scores.

Dependent Variable	Independent Variable	B	Standard Error_B_	Beta	*t*	*p*
Psychological Well-Being Scale (Block 1)	Fixed	15.384	4.743		3.244	0.001
	Empathy	0.112	0.056	0.088	1.997	0.046 *
	Quality of Sexual Life	0.068	0.016	0.197	4.188	0.000 **
	Relationship Satisfaction	0.376	0.055	0.324	6.882	0.000 **
R = 0.444 R^2^ = 0.197 Sd: 3/411 F = 33.654 *p* = 0.000 **						
Psychological Well-Being Scale (Block 2)	Fixed	3.364	4.999		0.673	0.501
	Empathy	0.143	0.054	0.112	2.662	0.008 **
	Quality of Sexual Life	0.064	0.016	0.186	4.122	0.000 **
	Relationship Satisfaction	0.372	0.053	0.320	7.084	0.000 **
	Relationship Status	−0.143	0.456	−0.017	−0.314	0.753
	Relationship Duration	0.791	0.568	0.065	1.393	0.164
	Number of Children	1.684	0.595	0.153	2.833	0.005 **
R = 0.541 R^2^ = 0.292 Sd: 8/406 F: 20.950 *p* = 0.000 **						

** *p* < 0.01. * *p* < 0.05.

## Data Availability

Data is contained within the article.
